# Supplementary material for: Excellence in Communication and Emergency Leadership (ExCEL): Pediatric First 5 Minutes Workshop for Residents
Source: MedEdPORTAL. 2020 Sep 25;16:10980. doi: 10.15766/mep_2374-8265.10980 (PMC7521066; doi:10.15766/mep_2374-8265.10980)
Supplement: Supplementary file 1 — First 5 Minutes Simulation.docxHigh-Quality CPR.pptxFirst 5 Minutes Workshop Evaluation Form.docx [file mep_2374-8265.10980-s001.zip › A. First 5 Minutes Simulation.docx]

| **Appendix A: First 5 Five Minutes Scenario**  **SIMULATION CASE TITLE:** ExCEL First 5 Minutes Scenario  AUTHORS: Robyn Wing, MD, MPH & Mariann Nocera Kelley, MD  LEARNER AUDIENCE: Pediatrics, Medicine-Pediatrics, Triple Board (pediatrics, psychiatry and child psychiatry), Family Medicine and Emergency Medicine residents. | |
| --- | --- |
| **PATIENT NAME:**  Maggie Brown  **PATIENT AGE:** 6 months old  **CHIEF COMPLAINT:** Altered mental status/Unresponsive  **PHYSICAL SETTING:** Pediatric hospital ward | |
|  | |
| **Brief narrative description of case** | Participants are informed that they are on the pediatric wards when the nurse for an infant admitted for bronchiolitis calls you into the patient room frantically to inform you that she is not waking up. Learner goals include to recognize and evaluate the unresponsive patient, perform basic life support (BLS) and mobilize the appropriate personnel and resources to manage a coding pediatric patient. |
| **Primary Learning Objectives** | 1.     Recognize pulseless patient and initiate Basic Life Support (BLS)  2.     Perform appropriate steps of the first five minutes of the management of a critically ill patient  3.     Demonstrate initial control of a critical situation with strong leadership  4.     Demonstrate high-quality cardiopulmonary resuscitation (CPR) |
| **Critical Actions** | 1. Check responsiveness 2. Check for a central pulse (pulse absent) 3. Call a “code” (institution specific) 4. Start high quality compressions with backboard and stool 5. Clear airway, bag-valve mask (BVM) ventilation, suction PRN 6. Put defibrillator pads onto patient and attach to defibrillator 7. Perform pulse and rhythm check simultaneously 8. Clarify patient’s weight 9. Obtain access – IV, IO |
| **Learner Preparation** | Assure learners of psychological safety during and after their participation. This brief simulation is intended to be formative and is solely for learning purposes.  The facilitators orient learners to the mannikin, demonstrating mannikin capabilities, and encourage learners to ask questions if they are unsure of something due to the simulation setting. Inform learners that they will be working as a team in caring for this patient.  The facilitators describe the setting for this scenario as follows:  *You are on the pediatric wards when the nurse for a 6-month-old infant admitted for bronchiolitis calls you into the patient room frantically to inform you that he is not waking up.* |

| Initial Presentation | | | | | |  |
| --- | --- | --- | --- | --- | --- | --- |
| **Initial vital signs** | | T 37.5°C  BP Unable to detect  HR 0  RR 0  O_2_ 60% on RA | | | | |
| **Overall Setting and Appearance** | | The patient appears unresponsive without any respiratory effort. No monitors are on the patient.  Parent/guardian is not present (if asked, they went to the hospital cafeteria). | | | | |
| **Confederates (e.g., standardized participants) and their roles in the room at case start** | | Nurse #1: Bedside Nurse: The nurse is appropriately concerned, but does not typically care for pediatric patients. The nurse is helpful and knowledgeable. However, only those interventions requested by the learner(s) should be performed.  Nurse #2: Arrives when a code is called and brings the code cart. The nurse is appropriately concerned, familiar with the code cart, helpful and knowledgeable about basic life support and pediatric advanced life support.  Facilitator: May be same person as nurse or a separate person if available. Ensures that equipment is running properly, that leads are attached in correct orientation on mannikin (such that Zoll is able to detect pads) and answers questions from the participants about the patient’s physical exam findings. The facilitator also observes the performance of the learners, provides feedback and instruction to the nurse to facilitate case progression, and facilitates the debriefing session. | | | | |
| **HPI** | | Information volunteered by bedside nurse:  Maddie is 6-month-old female admitted for bronchiolitis. She was admitted for frequent suctioning needs, but has not required high flow nasal cannula. The nurse states that the parents left just about 15 minutes ago to go to the cafeteria. She went to suction the baby and suddenly she didn’t seem to be breathing. She tried to stimulate her but the baby did not respond.  The following details will be revealed only when asked by the learner:   - The patient’s weight is 7 kg. - She was a former full term, healthy infant. | | | | |
| **Past Medical/Surgical History** | | **Medications** | **Allergies** | **Family History** | | |
| Former full-term infant | | None | None | None | | |
| **Physical Examination** | | | | | |  |
| **General** | Unresponsive infant | | | |  |  |
| **HEENT** | Patent airway, normocephalic, atraumatic, symmetric facies without dysmorphic features | | | |  |  |
| **Neck** | Supple, no lymphadenopathy | | | |  |  |
| **Lungs** | No respiratory effort (lungs coarse with bagging) | | | |  |  |
| **Cardiovascular** | No pulse and no heart rate detected by auscultation | | | |  |  |
| **Abdomen** | Soft no hepatosplenomegaly, no masses | | | |  |  |
| **Neurological** | Unresponsive | | | |  |  |
| **Skin** | Cool, pale | | | |  |  |
| **GU** | Normal female genitalia | | | |  |  |
| **Psychiatric** | Unresponsive | | | |  |  |

| Instructor Notes - Changes and CASE Branch Points  *This section should be a list with detailed description of each step than may happen during the case. If medications are given, what is the response? Do changes occur at certain time points? Should the nurse or other participant prompt the learners at given points? Should new actors or participants enter, and when? Are there specific things the patient will say or do at given times? There are a few examples given, but it is expected that most cases will have many more changes and potential branch points.* | | |
| --- | --- | --- |
| **Intervention / Time point** | **Change in Case** | **Additional Information** |
| Patient is identified to be pulseless and CPR is initiated | If no 🡪 | RN can ask, “Does he have a pulse?” |
| Team leader is identified and other roles assigned | If no 🡪 | RN can ask, “Who is the team leader?” and “Have we assigned roles yet?” |
| Team leader identifies patient weight and orders code dose epinephrine | If no 🡪 | RN can ask, “What is the patient weight?” and “Do you want me to draw up any meds?” |
| Patient placed on defibrillator | Defibrillator presents asystole rhythm | When learners direct attention to rhythm check, facilitator ends the case |
|  | If no 🡪 | RN can ask, “Who is putting the patient on the defibrillator?” |

**Ideal Scenario Flow**

The learners enter the room to find the patient unresponsive. They immediately check a central pulse and note the patient is pulseless. They start high quality CPR and instruct a team member to call a “code”. A team member calls a code (institution specific) and brings in code cart, step stool, backboard and defibrillator. One team member identifies self to the room as the “code leader”. He/she identifies the bedside nurse and medication nurse and instructs other physician team members on code team roles include documenter, chest compressors, airway physician, and CPR coach (or physician to manage the defibrillator if your institution does not utilize a CPR coach). The patient receives high quality CPR with a ratio of 15 compression to two breaths (2-rescuer CPR). Compressors are rotated every two minutes. Each bagged breath achieves good chest rise. The team leader identifies a patient weight and instructs the medication nurse to draw up multiple doses of code dose epinephrine. The team leader instructs the bedside nurse to obtain intravenous access or instructs a physician team member to obtain intraosseous access. The patient is placed on defibrillator pads and, under the clear instruction of the team leader, the team immediately pauses for a pulse and rhythm check. One team member identifies that the patient is still pulseless. The team leader looks at the rhythm and identifies it as an unshockable rhythm and instructs that CPR be continued. There is no longer than a ten second pause for the pulse and rhythm check or compressor rotations. The case ends at five minutes or after the first pulse and rhythm check (whichever comes first).

Anticipated Management Mistakes

Specific prompts and responses for anticipated management mistakes are outlined in the “Instructor notes- changes and branch points” section above. To summarize, they include:

- Failure to identify patient is pulseless. This is a pivotal action in the case. If learners do not identify that the patient is pulseless, the confederate RN may ask, “Does he have a pulse?” to prompt the action. This should be discussed in the debriefing.
- Failure to identify team leader and other team roles. If learners do not identify code team roles, the scenario may move forward in a disjointed way. Due to the short nature of the scenario, the confederate RN can prompt the learners by asking, “Who is the team leader?” and “Have we assigned roles yet?”. This should be discussed in the debriefing.
- High quality CPR is not delivered. This can occur in numerous ways, including incorrect ratio of compression to breaths, inadequate rate, inadequate depth or failure to achieve chest rise with BVM. Due to the short nature of the case, the RN can point out poor quality CPR with a prompt (“Isn’t the ratio for kids 15 to 2?” or “That CPR seems very fast to me.”) or this discussion point can be saved for the debrief.
- Failure of team to place patient on defibrillator monitor. This is a pivotal action in the case as the ultimate treatment of the patient depends on attaining a rhythm. If the learners do not place the defibrillator pads on the patient, the confederate RN may suggest it by asking “Who is placing the patient on the defibrillator?” This should also be discussed in the debriefing session after the simulation.

| Debriefing Guide: Adapted from the GAS model^1^  (Please refer to the Learning Objectives and Critical Actions to help guide discussion) | |
| --- | --- |
| **Debriefing Phase** | Suggested questions/phrases: |
| Transition into the debriefing: | That was a challenging/stressful/difficult case. Let’s talk about what just happened.  We moved through that scenario very quickly. Let’s talk about how things went. |
| Gather:  Shared Mental Model: Review facts of the case that just occurred. | - What happened when you first entered the room? - [Bill] did a lot of things very well in this case. What specifics did you notice that [Bill] did well? - Who were the members of the code team and how were roles established or assigned? - After [____], what happened next? - After [____], how did the team respond? |
| Analyze:  Learner-Centered Refection: Ask questions to understand learners’ thought process. | - I noticed you chose to [____], tell us about your thought process at that point. - What part of this scenario was particularly challenging? Why? - What elements of team communication went well? What could be improved upon? - What unanswered questions do you have about this case? |
| Summarize:  What are the take home points? | - What is your take home point? (go around the room and have every learner state one) - If you did this simulation again, is there anything you would do differently? |

References:

1. Phrampus P ODJ. Debriefing using a structured and supported approach. In: Levine A DS, Schwartz A, Sim A, ed. *The Comprehensive Textbook of Healthcare Simulation.* 1st ed. New York, NY: Springer; 2013:73-85.
