# Supplementary material for: Excellence in Communication and Emergency Leadership (ExCEL): Pediatric First 5 Minutes Workshop for Residents
Source: MedEdPORTAL. 2020 Sep 25;16:10980. doi: 10.15766/mep_2374-8265.10980 (PMC7521066; doi:10.15766/mep_2374-8265.10980)
Supplement: Supplementary file 1 — First 5 Minutes Simulation.docxHigh-Quality CPR.pptxFirst 5 Minutes Workshop Evaluation Form.docx [file mep_2374-8265.10980-s001.zip › B. High-Quality CPR.pptx]

## Slide 1
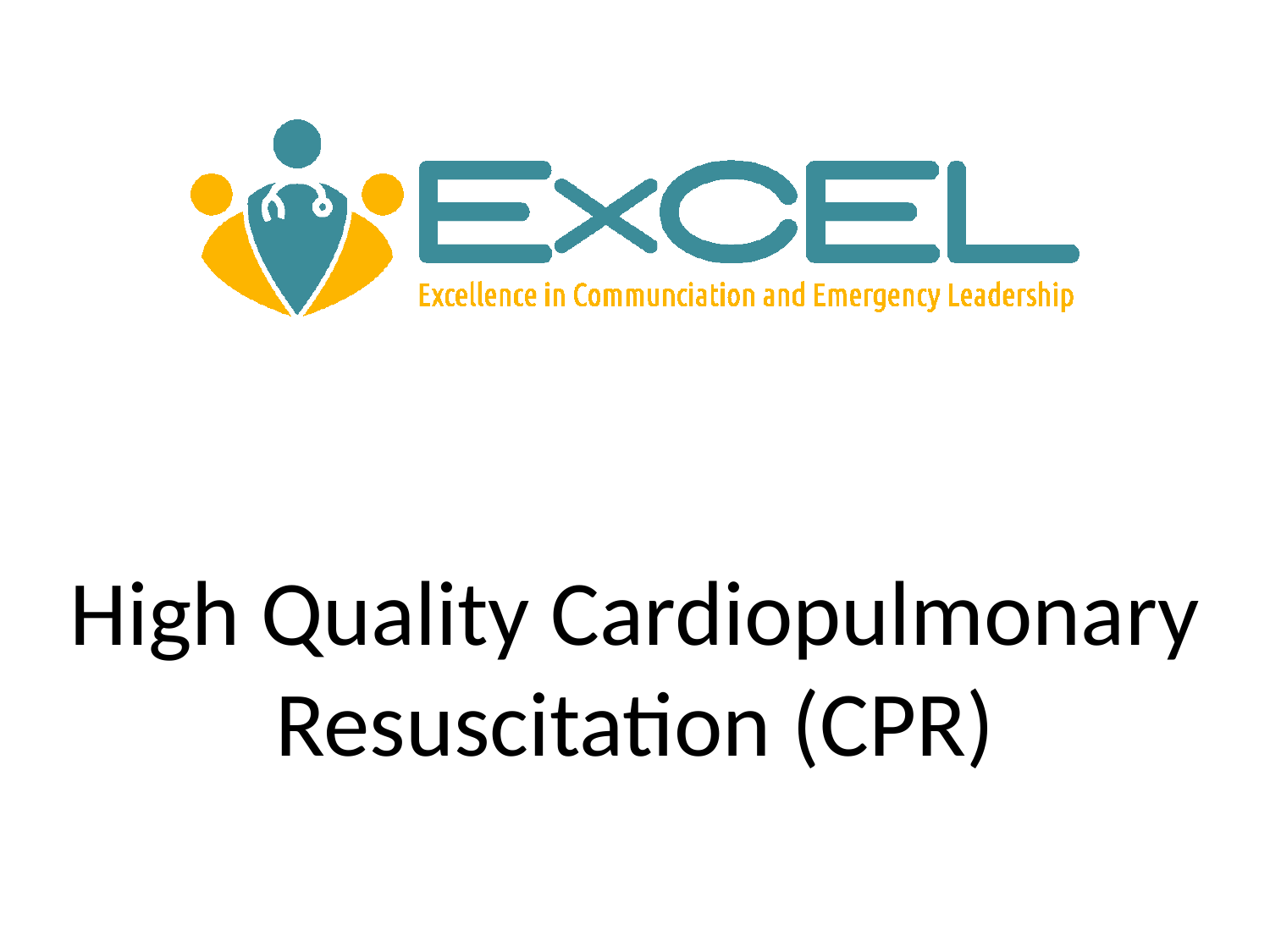

# High Quality Cardiopulmonary Resuscitation (CPR)

## Slide 2
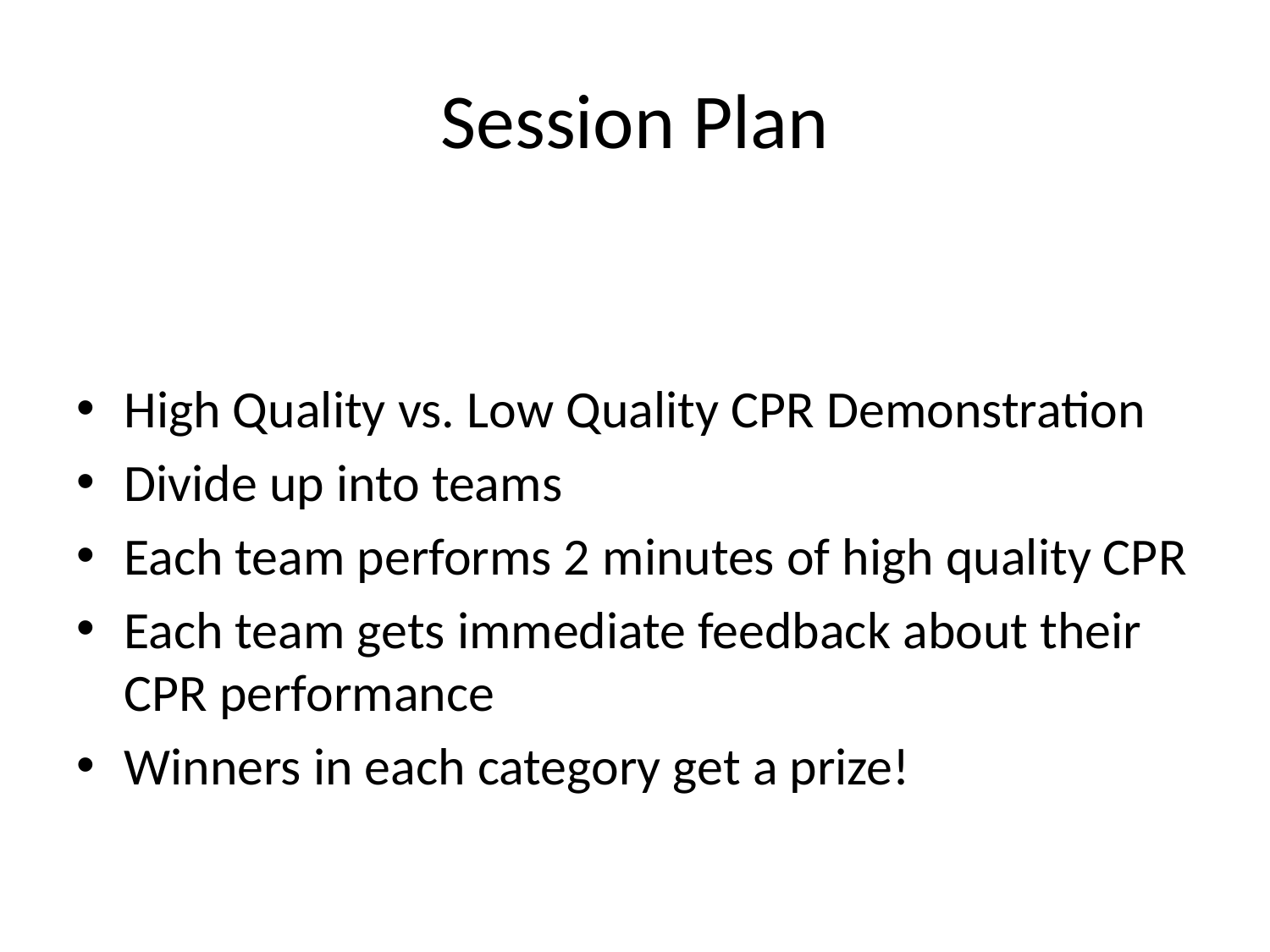

# Session Plan
High Quality vs. Low Quality CPR Demonstration
Divide up into teams
Each team performs 2 minutes of high quality CPR
Each team gets immediate feedback about their CPR performance
Winners in each category get a prize!

## Slide 3
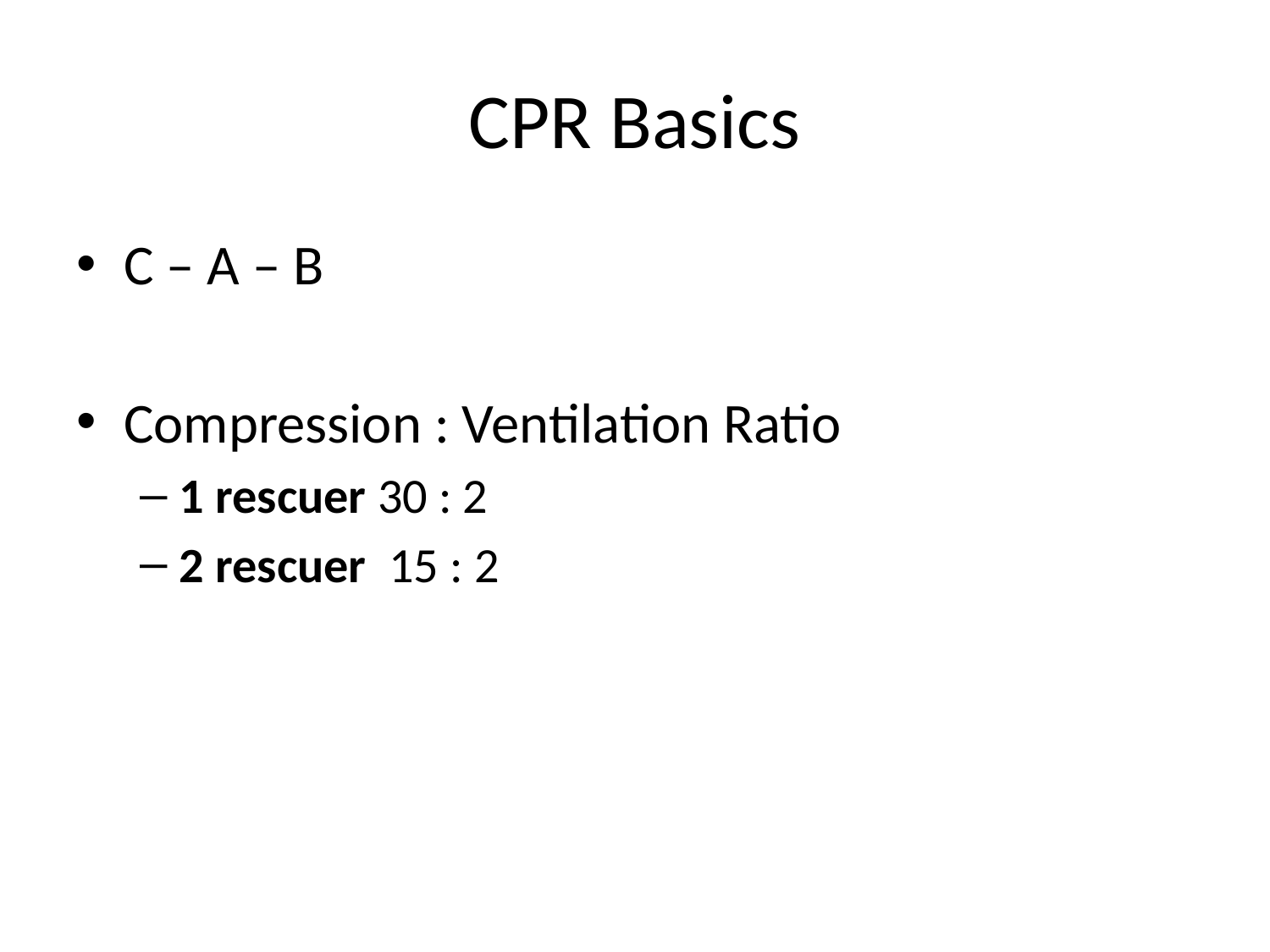

# CPR Basics
C – A – B
Compression : Ventilation Ratio
1 rescuer 30 : 2
2 rescuer 15 : 2

## Slide 4
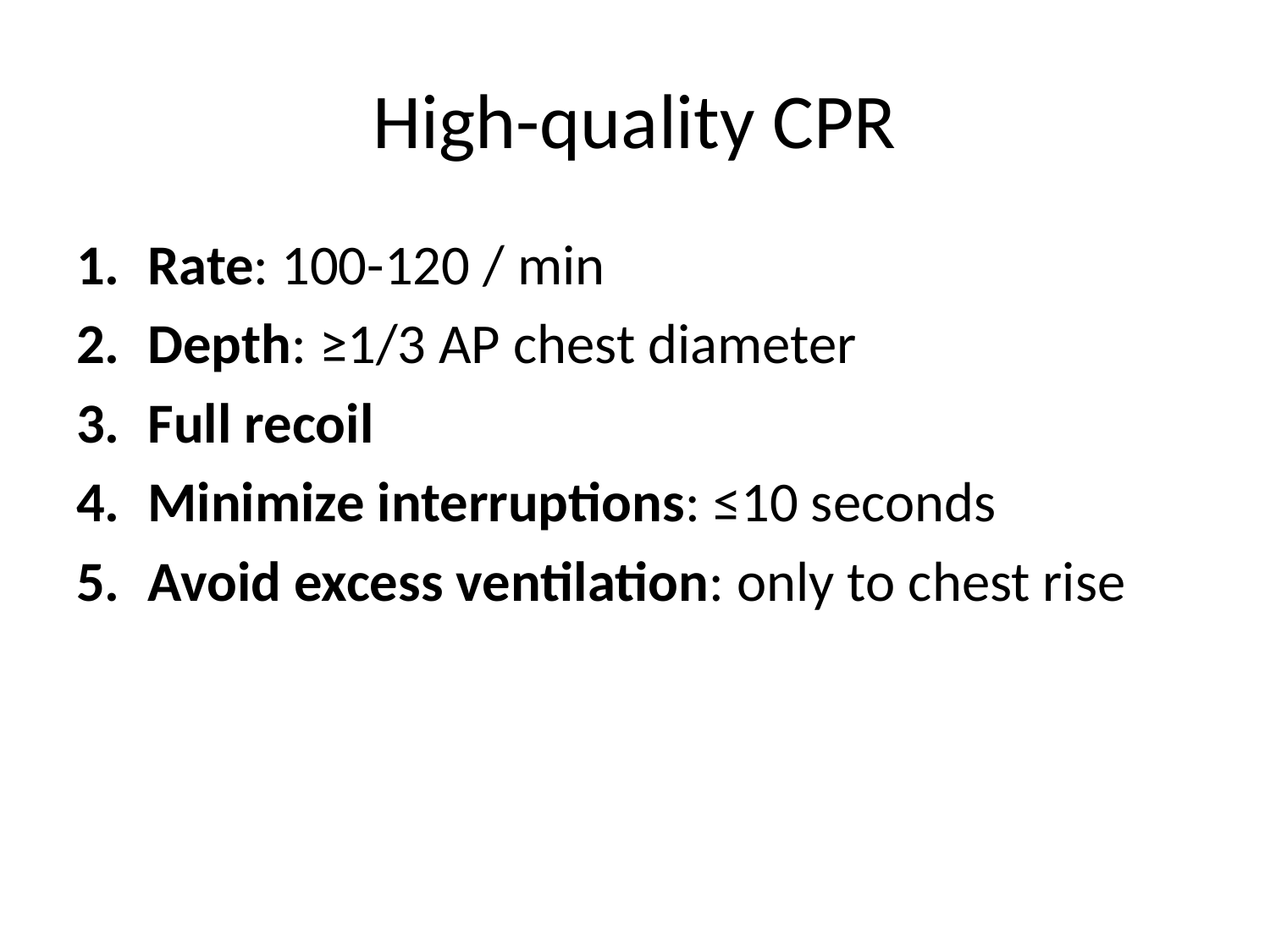

# High-quality CPR
Rate: 100-120 / min
Depth: ≥1/3 AP chest diameter
Full recoil
Minimize interruptions: ≤10 seconds
Avoid excess ventilation: only to chest rise

## Slide 5
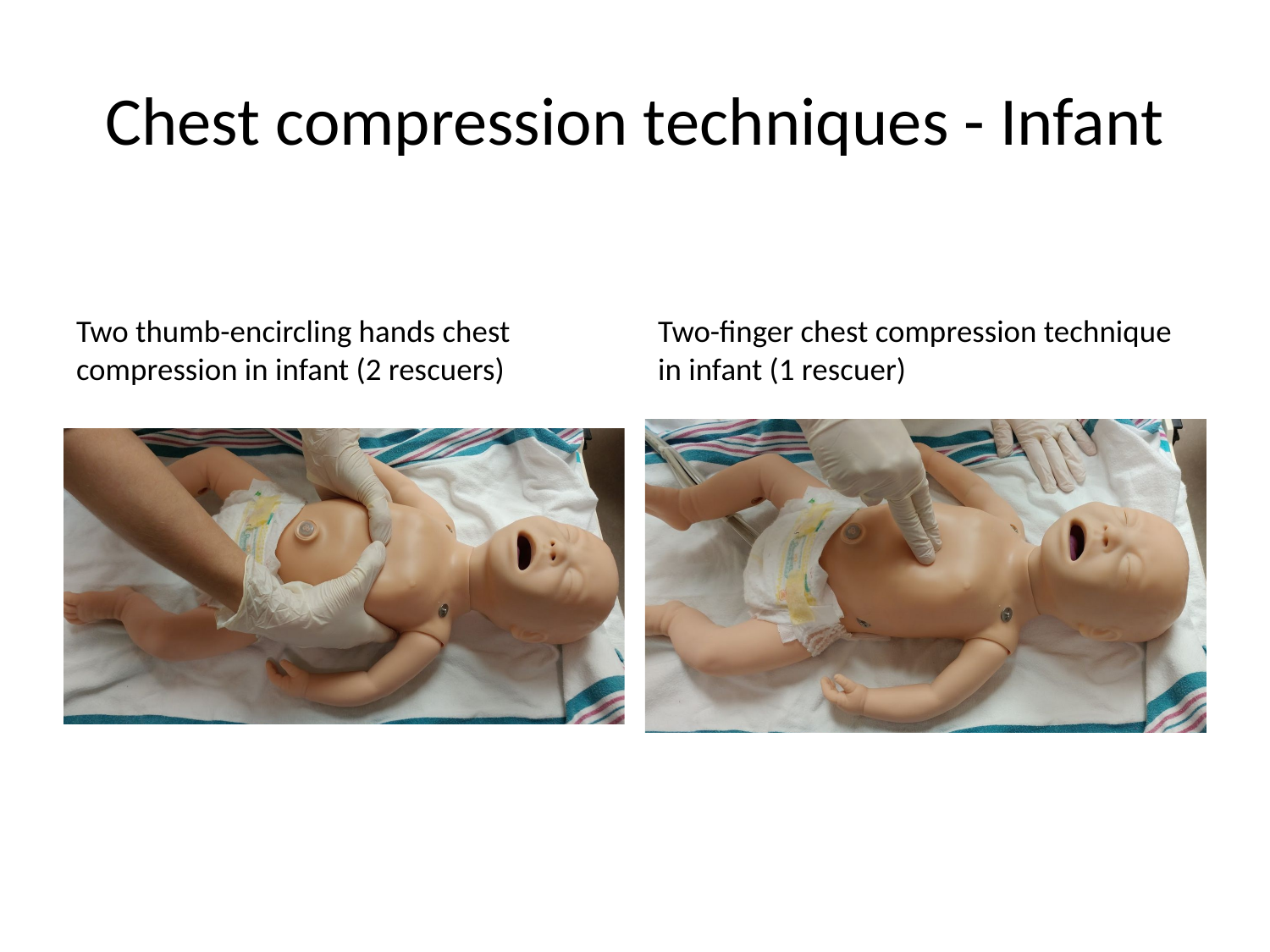

# Chest compression techniques - Infant
Two thumb-encircling hands chest compression in infant (2 rescuers)
Two-finger chest compression technique in infant (1 rescuer)

## Slide 6
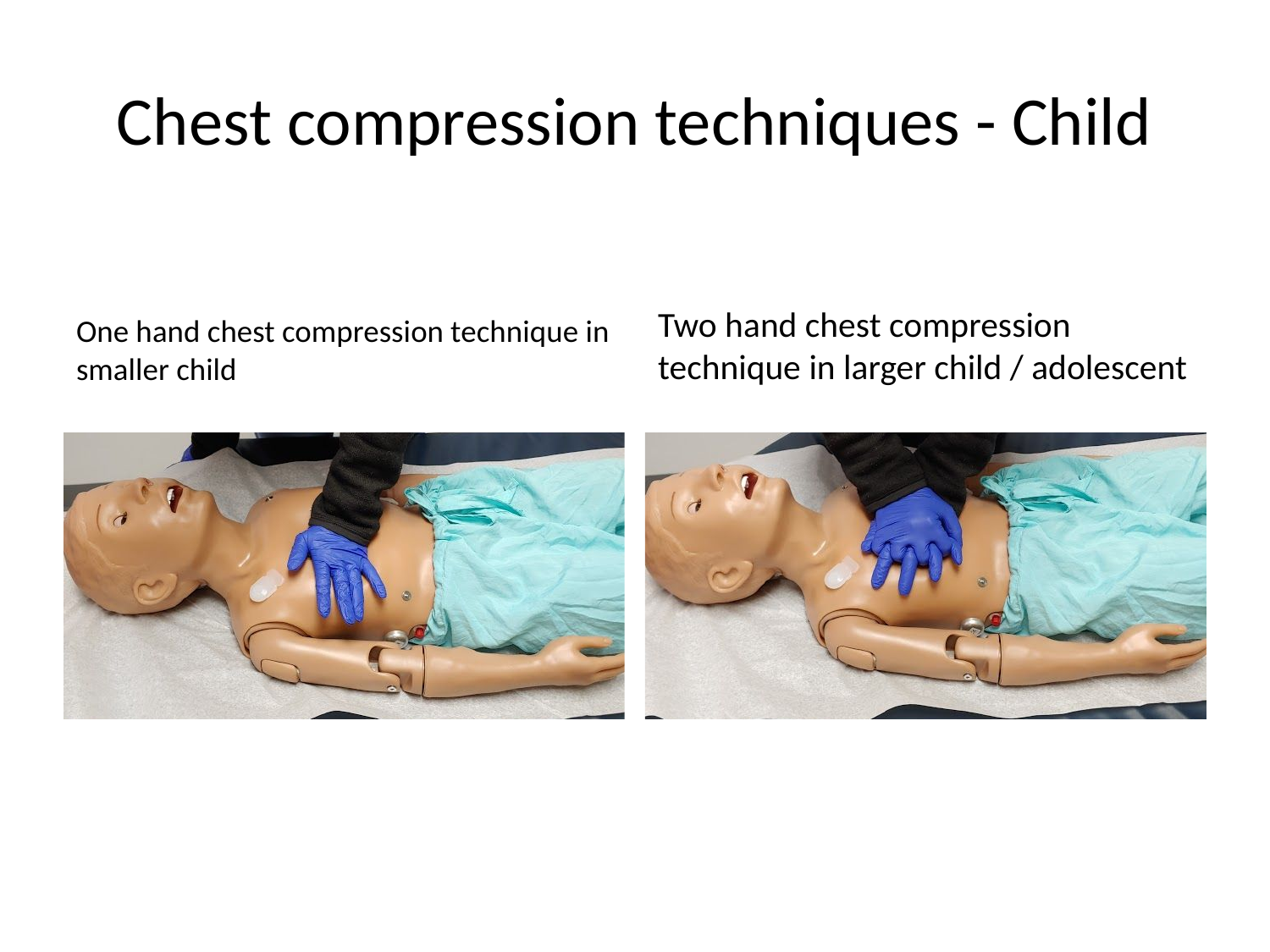

# Chest compression techniques - Child
Two hand chest compression technique in larger child / adolescent
One hand chest compression technique in smaller child
