# Supplementary material for: Excellence in Communication and Emergency Leadership (ExCEL): Pediatric First 5 Minutes Workshop for Residents
Source: MedEdPORTAL. 2020 Sep 25;16:10980. doi: 10.15766/mep_2374-8265.10980 (PMC7521066; doi:10.15766/mep_2374-8265.10980)
Supplement: Supplementary file 1 — First 5 Minutes Simulation.docxHigh-Quality CPR.pptxFirst 5 Minutes Workshop Evaluation Form.docx [file mep_2374-8265.10980-s001.zip › C. First 5 Minutes Workshop Evaluation Form.docx]

**ExCEL Survey – First 5 Minutes Workshop**

1. I am a:

☐ Pediatric Intern ☐ Pediatric Resident ☐ EM Intern ☐ EM Resident

☐ Family Medicine Resident ☐ Medical Student

☐ Other _______________________

1. Have you participated in this ExCEL skills session in the past?

☐ Yes ☐ No ☐ Unsure

3. When was the last time you took PALS?

☐ <6 months ☐ 6 months – less than 1 year ☐ 1-2 years ☐ Unsure

**Please rate your agreement with the following statements:**

|  |  | Strongly Disagree | Disagree | Neither Agree nor Disagree | Agree | Strongly Agree |
| --- | --- | --- | --- | --- | --- | --- |
| 4. | This workshop was relevant to my work. | □ | □ | □ | □ | □ |
| 5. | This workshop was effective in teaching me the assessment and management of a critical patient in the first 5 minutes of a critical situation. | □ | □ | □ | □ | □ |
| 6. | This workshop was effective in teaching me how to perform high-quality CPR. | □ | □ | □ | □ | □ |

**After participating in this workshop how confident are you in your ability to:**

|  |  | Very Not confident | Not confident | Neutral | Confident | Very Confident |
| --- | --- | --- | --- | --- | --- | --- |
| 7. | Initially manage a critical situation? | □ | □ | □ | □ | □ |
| 8. | Assess and emergently manage airway, breathing, and circulation in the first 5 minutes of a critically ill patient? | □ | □ | □ | □ | □ |
| 9. | Describe the markers of high-quality CPR? | □ | □ | □ | □ | □ |
| 10. | Perform high-quality CPR? | □ | □ | □ | □ | □ |

11. What did you find most helpful about this workshop?

12. What do you think could be improved upon for this workshop in the future?

13. Other comments or suggestions:
