# Supplementary material for: Excellence in Communication and Emergency Leadership (ExCEL): Pediatric First 5 Minutes Workshop for Residents
Source: MedEdPORTAL. 2020 Sep 25;16:10980. doi: 10.15766/mep_2374-8265.10980 (PMC7521066; doi:10.15766/mep_2374-8265.10980)
Supplement: Supplementary file 1 — First 5 Minutes Simulation.docxHigh-Quality CPR.pptxFirst 5 Minutes Workshop Evaluation Form.docx [file mep_2374-8265.10980-s001.zip › _Educational_Summary_Report_10980.pdf]

MedEdPORTAL® The AAMC Journal of Teaching and Learning Resources

Original Publication Open Access

Excellence in Communication and Emergency Leadership (ExCEL): Pediatric First 5 Minutes Workshop for Residents

Robyn Wing, MD, MPH*, Hoi See Tsao, MD, Vanessa Toomey, MD, MBA, Laura Mercurio, MD, Marie Carillo, MD, Linda L. Brown, MD, MSCE, Mariann Nocera Kelley, MD

*Corresponding Author: rwing901@gmail.com

Abstract

Introduction: In-hospital pediatric cardiopulmonary arrest is associated with high morbidity and mortality, and appropriate initial management has been associated with improved clinical outcomes. Despite current training, pediatric residents often do not feel confident in their ability to deliver this initial management. This workshop focused on the initial management of critically ill pediatric patients and performance of high-quality CPR. **Methods:** This hands-on workshop utilized skill stations with low- and medium-fidelity simulators to instruct learners on initial management during the first 5 minutes of a code, including high-quality CPR. It was designed for residents across all levels of training who care for pediatric patients (including pediatrics, medicine-pediatrics, pediatrics, psychiatry, and child psychiatry, family medicine, and emergency medicine residents) and can be adapted for different session durations and group sizes. **Results:** This workshop was conducted at two separate institutions with a total of 18 resident participants. Participants strongly agreed that this workshop was relevant and effective in teaching the initial assessment and management of the critical pediatric patient, including how to best perform high-quality CPR. Residents further reported high levels of confidence in initially assessing and managing a critically ill patient, describing the markers of high-quality CPR, and performing high-quality CPR. **Discussion:** This workshop provided residents with additional instruction and practice in the initial management of critically ill pediatric patients in cardiopulmonary arrest. The structure and timeline of this curriculum can be adapted to the needs of the individual institution's program and the number of workshop participants.

Keywords

Pediatric Residents, Leadership, Cardiopulmonary Resuscitation, Pediatric Critical Care Medicine, Pediatric Emergency Medicine, Case-Based Learning, Clinical/Procedural Skills Training, Simulation

Educational Objectives

By the end of this activity, learners will be able to:

1. Demonstrate initial control of a critical situation with strong leadership.
2. Perform appropriate steps of the first 5 minutes of the management of a critically ill patient.
3. Identify metrics of high-quality CPR.
4. Demonstrate high-quality CPR.

Introduction

Cardiopulmonary arrest occurs in 0.1% to 3% of children admitted to the hospital[1] and has high morbidity and mortality, with survival rates ranging from 14% to 36%.[2,3] The first 5 minutes in a resuscitation is an especially critical time due to the potential for clinical improvement prior to irreversible deterioration. Patients who receive CPR within 1 minute of collapse are more than twice as likely to survive.[4] CPR initiation within 3 minutes of cardiac arrest is associated with a 25% increase in survival.[5] Despite the low frequency of pediatric cardiopulmonary arrests, almost all pediatric residents are involved in a cardiopulmonary arrest resuscitation at least once during their training.[1] It is therefore critical that pediatric residents are proficient at managing the first minutes of a cardiopulmonary arrest to maximize patient survival and outcomes.

Citation:
Wing R, Tsao HS, Toomey V, et al. Excellence in Communication and Emergency Leadership (ExCEL): pediatric first 5 minutes workshop for residents. *MedEdPORTAL*. 2020;16:10980.
https://doi.org/10.15766/mep_2374-8265.10980

Although most pediatric residents have received resuscitation training through the pediatric advanced life support (PALS) curriculum, translation of this training to patient care is poor. PALS alone inadequately prepares pediatric residents for resuscitations, as residents are unable to perform skills such as airway assessment and management, rhythm recognition,

Copyright © 2020 Wing et al. This is an open-access publication distributed under the terms of the Creative Commons Attribution license.

and defibrillation consistently and independently after PALS training.[1,6,7] Even if pediatric residents are initially proficient, they may lose resuscitation skills as soon as 8 months after initial resuscitation training, eroding the quality of the resuscitation and the confidence of residents leading them.[8] This indicates a need for periodic refreshers of knowledge and skills. In terms of confidence, one study showed that less than half of third-year pediatric residents felt confident in their ability to lead resuscitations, with 89% reporting the need for further training to achieve competence in gaining the technical skills needed for resuscitation.[9]

Regardless of practice choice, all graduating pediatric residents will likely encounter acutely ill children during their careers. Given the low frequency of pediatric codes, patient simulators have become an increasingly important part of training for medical residents to maintain high-level resuscitation skills.[8,10] Simulation-based education interventions reduce the time to call for help, initiate bag-mask ventilation (BVM), and start CPR for first-year pediatric residents.[11] Participants and observants in mock code programs have also reported increased self-confidence and comfort with knowledge during a code, as well as decreased anxiety.[12,13] Promisingly, an increased number of mock codes at one tertiary care academic medical center has been associated with improved survival rates from 33% to 50% in pediatric cardiopulmonary arrests.[14]

This workshop is the first in a series from our larger curriculum titled *Excellence in Communication and Emergency Leadership*, or ExCEL. The ExCEL curriculum was developed with a goal to replace standard resident morning report didactics once a month with simulation-based training and core resuscitation skills sessions. Data from unannounced multidisciplinary in situ pediatric mock resuscitations at our institution identified specific areas for improvement including team leadership and communication skills, timely and accurate rhythm recognition for patients in cardiac arrest, and performance of quality CPR. The ExCEL curriculum aimed to augment the skills initially acquired during PALS courses by helping residents to gain confidence, sharpen and maintain critical technical skills, and improve leadership and communication proficiency. The monthly small-group workshops incorporated case-based learning, active commitment exercises, and hands-on practice of technical skills including, but not limited to: airway skills, CPR with quality chest compressions, and the use of defibrillators. This first 5 minutes workshop may be used independently or in conjunction with other sessions from the ExCEL curriculum.

Given the importance of the first 5 minutes of management in improving outcomes in children with cardiopulmonary arrest, we have developed a novel curriculum to enhance pediatric residents' ability to perform the critical steps during this timeframe. While another study has referenced a simulation-based approach to teaching first 5 minutes resuscitation skills,[15] to our knowledge there is not a similar workshop published in *MedEdPORTAL* to guide institutions on implementing such a curriculum. This previously published study[15] utilized only simulation cases with a rapid cycle deliberate practice (RCDP) curriculum style, while our curriculum utilized small-group breakout sessions including a simulation case and a CPR skills session. The primary goal of this workshop was to prepare residents to successfully manage the first 5 minutes of a critically ill patient's resuscitation. This curriculum can be repeated at regular intervals throughout the academic year to reinforce knowledge and skills, increase comfort with pediatric resuscitations, and ideally translate into improved patient care.

Methods

Target Audience

The target audience for our curriculum included pediatrics, medicine-pediatrics, triple board (pediatrics, psychiatry, and child psychiatry), and family medicine and emergency medicine residents (PGY 1-5). Other learners that often attended these educational sessions included rotating third- and fourth-year medical students, but these learners were not our target learners. We ran this workshop at two different institutions, adapting the workshop to the needs and structure of the morning report at each institution.

Instructor/Facilitator

There were two sections to this workshop which required separate instructors to run them simultaneously.

First 5 minutes simulation session (Appendix A): The instructors for this session were faculty members, fellows, or chief residents who possessed both knowledge of basic life support (BLS) and PALS, as well as training in simulation and medical education. It was also imperative that instructors had significant experience and knowledge of the resources and systems in place for pediatric code management at their specific institution. This breakout session required at least one instructor who acted as both a facilitator and nurse actor in a very brief simulation. When possible, two instructors allowed for a separate facilitator and nurse actor.

CPR Challenge session (Appendix B): The instructors for this session possessed knowledge and experience with pediatric

Copyright © 2020 Wing et al. This is an open-access publication distributed under the terms of the Creative Commons Attribution license.

Table 1. Suggested Timeline for First 5 Minutes Workshop (30 or 60 minutes)

Item	Activity	Time (60-minute workshop)	Time (30-minute workshop)
Introduction	Introduction/breaking into small groups	5 min.	5 min.
Skills session one	CPR challenge or first 5 minutes simulation	25 min.	10 min.
Skills session two	CPR challenge or first 5 minutes simulation	25 min.	10 min.
Question and answer, and workshop evaluation	Large-group discussionComplete evaluation	5 min.	5 min.

BLS and high-quality CPR as well as the ability to operate the CPR feedback device. This included simulation technicians, pediatric emergency medicine attendings or fellows, and pediatric chief residents. This session required at least one instructor to run the mannequin and provide CPR feedback to participants.

Setting

When the two breakout sessions occurred simultaneously, this workshop took place in two separate learning areas such that each skills session had its own space in order to facilitate optimal learning. This was done in two nearby patient rooms, adjacent conference rooms, or in a large conference room with partitioning. Learners were divided into two groups: one group started in the first 5 minutes simulation session and the other started in the CPR challenge skills session.

Timeline

Given the hands-on nature of this workshop, the time necessary for completion depended upon the number of learners participating. At one institution, a 60-minute timeframe was necessary due to constraints on educational time in our learners' clinical day, as our sessions took place during typical morning report hours. This timeframe allowed for up to 24 participants, split into two groups of 12 learners. However, this workshop timeline could be adapted to accommodate for more learners. At a second institution with a smaller number of learners and facilitators, this workshop was completed in a 30-minute timeframe, with the first 5 minutes simulation session and CPR challenge session occurring sequentially. For more details, see Table 1.

The total workshop duration depended on the number of participants in attendance. For the CPR challenge session, the 60-minute workshop format allowed for up to 12 participants to have 2 minutes for CPR practice and 1 minute for CPR competition each, all within 25 minutes. The 30-minute workshop format allowed four participants the same opportunities within 10 minutes as they could rotate through more quickly.

For the first 5 minutes simulation session, the 60-minute workshop format allowed for three groups of three to four participants each the opportunity to participate in a 5-minute simulation scenario. In the 30-minute workshop format, a single group of up to four participants participated in the 5-minute simulation scenario allowing for a shorter duration of this session and the workshop overall.

Equipment

In advance of the workshop, instructors identified and secured the necessary equipment for this workshop, including the items listed in Table 2.

Set-up

On the day of the workshop, instructors arrived early for set-up and testing of each mannequin and CPR feedback device to ensure proper functioning.

First 5 minutes simulation session room: The room was set up with the mannequin on a stretcher (or a table, if a stretcher was not available) programmed with an asystole rhythm. The scenario could be adapted to involve other rhythms based on the particular goals and objectives of the session. However, our goals did not include rhythm recognition. The fully stocked code cart was present off to the side of the room along with the defibrillator. If a backboard and stepstool were not part of the code cart, these items were also available off to the side of the room as well.

Table 2. Equipment List for First 5 Minutes Workshop

First 5 Minutes Simulation Session	CPR Challenge Skills Session
Medium-fidelity mannequin (i.e., Sim Junior)[a]	Low-fidelity mannequin or CPR task trainer(s)[b]
Backboard	CPR feedback device (e.g., Zoll R series, qCPR meter)
Stepstool	Backboard
Code cart	Stepstool
Bag-mask ventilation (BVM) with mask	BVM with mask
Defibrillator	Defibrillator
Stretcher	Whiteboard and marker

[a]Mannequin that allows for placement of airway adjuncts, assessment of chest rise with BVM, CPR, and cardiac rhythm generation.
[b]Mannequin that allows for assessment of chest rise with BVM and CPR feedback. We used Little Anne qCPR mannequin with qCRP instructor application from Laerdal. However, other similar systems would be adequate.

Copyright © 2020 Wing et al. This is an open-access publication distributed under the terms of the Creative Commons Attribution license.

CPR Challenge skills session room: The room was set up with either the mannequins or CPR task trainers in the middle of the room on a table.

First 5 Minutes Simulation Session Content

Participants were separated into groups of three to four with mixed training levels. The scenario (as outlined in the simulation scenario in Appendix A) began by telling the participants that they were on the pediatric wards when the nurse for an infant admitted for bronchiolitis calls them into the patient room frantically to inform them that he is not waking up. Participants were expected to begin the evaluation of an infant, starting with BLS procedures. The patient was unresponsive and had no pulse or respiratory effort. Participants were expected to successfully complete the critical actions of the first 5 minutes of a pediatric code including assessment of airway, breathing and circulation, airway maneuvers, BVM ventilation, defibrillator attachment, and high-quality CPR. After 5 minutes (or after the first pulse and rhythm check), the case was ended by the facilitator.

The sessions that included up to 12 participants ran each 5-minute scenario several times, to allow each group of three or four learners the opportunity to participate in the simulation exercise. Therefore, this necessitated a longer time for this session of the workshop and the workshop in general. The shorter workshop durations (30 minutes) included only one group of three or four learners and therefore the simulation exercise ran only one single time with all learners participating.

Debrief: The case scenario was then debriefed by the facilitators. As an interactive, bidirectional, and reflective discussion, debriefing is the most important feature of simulation-based education.[16] The primary debriefing method in ExCEL included the three-phase conversational structured debrief known as the gather-analyze-summarize (GAS) model.[17] Adopted by the American Heart Association for use during debriefing in PALS courses, the GAS model provides a simple yet complete structure for efficient debriefing.[18,19] The phases included gathering information regarding the facts of the case (creating a shared mental model), analyzing the learners' thought processes (learner-centered reflection), and summarizing take home points (Appendix A). Other participant groups within the same session who observed each simulated vignette were also invited to take part in the debrief. For larger groups, the simulation was repeated to ensure participation of all group members.

CPR Challenge Skills Session Content

The instructor led a brief discussion about the metrics for high-quality CPR using a brief presentation (Appendix B) and demonstrated the performance of both high-quality and low-quality CPR on the mannequin. Participants were paired with a partner for the CPR challenge. Each pair was allowed to practice CPR on the mannequin with a feedback device (such as the Zoll R series) so that they could see their CPR quality and make necessary improvements (for example, increase their rate or increase their depth). Each pair was then given a chance to compete with other pairs in performing high-quality CPR for 2 minutes (1 minute per participant). The facilitator then informed each group of their results for the following metrics: percent of compression in target rate range, percent of compressions in target depth range, overall percent of compressions in both target depth and rate, and percentage of time performing compressions. Use of multiple mannequins and feedback devices allowed for simultaneous practice and testing, with one faculty member monitoring the participants in the competition portion. If group size was sufficiently large, the top three pairs' scores for each category were recorded on a whiteboard for subsequent groups to see the statistics for the highest performing groups. The winning team received a small prize.

ExCEL Curriculum

As previously mentioned, this workshop is the first in a series from our larger ExCEL curricula. As an unordered series, this workshop can be given at any time in the curricula. The workshops are currently planned 6 months in advance but can be adjusted accordingly if facilitators identify a clinical concern on a relevant topic that could benefit from an educational intervention.

Evaluation

Residents were asked to complete the ExCEL first 5 minutes workshop evaluation form (Appendix C) as an evaluation of the workshop and to obtain feedback on our goals and objectives, as perceived by the learners on a 5-point Likert scale (1 = *strongly disagree*, 5 = *strongly agree*). This form also solicited feedback about participants' suggestions for improvement of the session. For ease of use and to maximize response rates, we converted this survey into an electronic form that was easily accessible with a QR code.

Results

This workshop was conducted at two separate institutions as part of the ExCEL curriculum with a total of 18 resident participants. The workshop length and format were adapted to the needs and structure of the morning report at each institution. At the first institution, the 60-minute workshop included 15 resident participants and four facilitators. The first 5 minutes and the CPR challenge scenarios occurred simultaneously at separate stations

Copyright © 2020 Wing et al. This is an open-access publication distributed under the terms of the Creative Commons Attribution license.

(Table 1). The resident participants were split into two groups of approximately seven to eight residents with two facilitators at each station. At the second institution, the workshop included three resident participants and one facilitator. The session ran over 30 minutes with the first 5 minutes simulation session and CPR challenge occurring sequentially (Table 1).

Of the 18 total participants, nine (50%) were interns and nine (50%) were residents. Eleven (61%) of the participants completed a PALS course within the preceding 6 months; the remainder reported taking the PALS course 1-2 years prior to the session. Based on our evaluation form, we received very positive feedback about learner satisfaction and self-efficacy to perform the tasks in the educational objectives (Tables 3 and 4).

Residents reported feeling that this workshop was effective in teaching the assessment and management of a critically ill patient in the first 5 minutes, as well as how to perform high-quality CPR (Table 3). Residents also reported feeling confident in their ability to manage the first 5 minutes of a critical situation and describe and perform high-quality CPR (Table 4). Further examination of responses revealed that the mean confidence scores for interns regarding their confidence to manage a critical situation and to assess and emergently manage airway, breathing, and circulation in the first minutes were lower than those of the senior residents ($M = 3.2$ vs. $M = 3.8$, and $M = 3.3$ vs. $M = 3.9$, respectively).

In the survey, participants wrote that they found the ability to practice in a scenario, use the equipment, and receive immediate feedback most helpful. Facilitators observed a positive trend in residents identifying themselves as code leader, performing high-quality CPR, and instructing their team on the initial steps to effectively manage a critically ill pediatric patient in subsequent workshops and simulations in the curriculum, as well as in hospital-wide unannounced mock codes. Of note, several participants suggested adding an example of a perfect first 5 minutes demonstration by the facilitators to demonstrate ideal management.

Table 3. Participant Feedback on First 5 Minutes Workshop

Statement	*M* (*n* = 18)[a]
This workshop was relevant to my work.	4.9
This workshop was effective in teaching me the assessment and management of a critical patient in the first 5 minutes of a critical situation.	4.8
This workshop was effective in teaching me how to perform high-quality CPR.	4.9

[a]Rated on a 5-point Likert scale (1 = *strongly disagree*, 2 = *disagree*, 3 = *neither agree nor disagree*, 4 = *agree*, 5 = *strongly agree*).

Table 4. Participant Feedback on Self-Confidence Related to Learning Objectives (*n* = 18)

Statement	*M*[a]	Percent of Respondents Answering 4 or 5[a]
After participating in this workshop, how confident are you in your ability to:		
Initially manage a critical situation?	3.5	50%
Assess and emergently manage airway, breathing, and circulation in the first 5 minutes of a critically ill patient?	3.6	61%
Describe the markers of high-quality CPR?	4.6	100%
Perform high-quality CPR?	4.1	83%

[a]Rated on a 5-point Likert scale (1 = *very not confident*, 2 = *not confident*, 3 = *neutral*, 4 = *confident*, 5 = *very confident*).

Discussion

High-quality CPR is a key intervention in reducing the high morbidity and mortality associated with cardiopulmonary arrest in children.[2-5] Most residents undergo PALS certification and recertification courses as part of their residency programs, though the further they are from this training, the less effective they are at managing critically ill patients.[8] The *Pediatric First 5 Minutes Workshop for Residents* was a relevant and effective educational intervention for residents. The skills reinforced in this workshop—the initial assessment and management of a critically ill child and the performance of high-quality CPR—are vital skills required for residents caring for this population.

Our data supported the existing literature that PALS alone inadequately prepares residents for the management of critically ill children.[6] In one study, the majority of senior pediatric residents reported that they did not feel confident leading resuscitations and the vast majority felt that they needed further training.[9] Our data showed that, while more than half of the participants had completed a PALS course in the preceding 6 months, they still did not feel entirely comfortable managing critically ill patients. After completion of our workshop, half of our participants felt confident in their ability to manage the initial few minutes of a critical patient and the majority felt confident in assessing and emergently managing the patient's airway, breathing, and circulation and performing high-quality CPR. This curriculum utilized small-group and hands-on learning, with an element of gamification in education in the CPR competition to keep learners actively engaged.

With each workshop iteration, we learned that the format and equipment needs of the session were determined by the number of resident participants. The ideal instructor-to-student ratio was about one instructor for every three to five residents to allow for adequate observation of learner actions and real-time feedback.

Copyright © 2020 Wing et al. This is an open-access publication distributed under the terms of the Creative Commons Attribution license.

For larger groups of residents, we found that the 60-minute format is ideal, but required a greater investment of resources including facilitators, equipment, and space. The extended session allowed for sufficient time to run multiple first 5 minutes simulation sessions, which residents reported was beneficial to their overall learning. The 30-minute session allowed for instruction of this workshop by only one facilitator, but left less time for running multiple first 5 minutes simulation sessions. Our experience with workshop length demonstrated that this workshop can be adapted to longer sessions, as dictated by the structure of an institution's program, the number of workshop participants, and the number of instructors and equipment. For example, if a longer time period is available, groups may participate in the simulation exercise more than once, allowing different members to act as team leaders.

A key limitation of our workshop was the diversity of participants' residency training experiences and their levels of training. Residents with different background training from their varying residency programs (pediatrics, medicine-pediatrics, triple board, family medicine vs. emergency medicine) may have had different familiarity and comfort with caring for critically ill children before attending our workshop. Therefore, this workshop may be of varying educational utility for residents in different specialties. Furthermore, while the varied session duration provided a slightly different experience for the participants at different institutions, their feedback supported this workshop's adaptability to fit the needs of individual institutions. Finally, this workshop was not designed to reteach all the concepts introduced in PALS certification and recertification courses, and therefore could not provide a comprehensive course on the resuscitation of critically ill children. It can, however, provide a supplement to the formalized training provided to residents as part of their regular didactic educational curriculum.

This workshop was designed as part of a larger curriculum, *Excellence in Communication and Emergency Leadership (ExCEL)*, and therefore can be implemented as a component of a larger morning report curriculum, or it can stand on its own as an independent educational tool. This workshop augmented PALS training by providing a guided opportunity for trainees to refresh their PALS skills between recertification sessions as well as a systematic framework to approach the first few minutes of managing a critically ill pediatric patient. Preliminary findings show that this ongoing simulation and case-based training opportunity can improve resident performance, including critical communication and leadership skills. Additional quantitative and qualitative data from both mock and actual pediatric resuscitations are currently being reviewed to further evaluate and optimize this curriculum. Based on resident feedback, the next iteration of this workshop will incorporate an instructor-demonstrated first 5 minutes of a code for residents to observe prior to their participation in the simulated scenario.

Appendices

A. First 5 Minutes Simulation.docx

B. High-Quality CPR.pptx

C. First 5 Minutes Workshop Evaluation Form.docx

All appendices are peer reviewed as integral parts of the Original Publication.

Robyn Wing, MD, MPH: Assistant Professor, Departments of Emergency Medicine and Pediatrics, Division of Pediatric Emergency Medicine, Alpert Medical School of Brown University and Rhode Island Hospital/Hasbro Children's Hospital; Director of Pediatric Simulation, Lifespan Medical Simulation Center

Hoi See Tsao, MD: Fellow, Departments of Emergency Medicine and Pediatrics, Division of Pediatric Emergency Medicine, The Alpert Medical School of Brown University and Rhode Island Hospital/Hasbro Children's Hospital

Vanessa Toomey, MD, MBA: Clinical Fellow, The Department of Anesthesiology, Critical Care, and Pain Medicine, Harvard Medical School and Boston Children's Hospital

Laura Mercurio, MD: Fellow, Departments of Emergency Medicine and Pediatrics, Division of Pediatric Emergency Medicine, The Alpert Medical School of Brown University and Rhode Island Hospital/Hasbro Children's Hospital

Marie Carillo, MD: Fellow, Department of Cardiology, Children's National Medical Center

Linda L. Brown, MD, MSCE: Associate Professor, Departments of Emergency Medicine and Pediatrics, Division of Pediatric Emergency Medicine, Alpert Medical School of Brown University and Rhode Island Hospital/Hasbro Children's Hospital; Director of the Lifespan Medical Simulation Center

Mariann Nocera Kelley, MD: Assistant Professor, Departments of Pediatrics and Emergency Medicine, Division of Pediatric Emergency Medicine, University of Connecticut School of Medicine, Connecticut Children's; Director of Simulation, University of Connecticut School of Medicine

Acknowledgments
Drs. Linda L. Brown and Mariann Nocera Kelley are co-senior authors.

Disclosures
None to report.

Copyright © 2020 Wing et al. This is an open-access publication distributed under the terms of the Creative Commons Attribution license.

Funding/Support
None to report.

Ethical Approval
Reported as not applicable.

References

1 Hunt EA, Patel S, Vera K, Shaffner DH, Pronovost PJ. Survey of pediatric resident experiences with resuscitation training and attendance at actual cardiopulmonary arrests. *Pediatr Crit Care Med*. 2009;10(1):96-105. https://doi.org/10.1097/PCC.0b013e3181937170

2. Hunt EA, Walker AR, Shaffner DH, Miller MR, Pronovost PJ. Simulation of in-hospital pediatric medical emergencies and cardiopulmonary arrests: highlighting the importance of the first 5 minutes. *Pediatrics*. 2008;121(1):e34-e43. https://doi.org/10.1542/peds.2007-0029

3. Couloures KG, Allen C. Use of simulation to improve cardiopulmonary resuscitation performance and code team communication for pediatric residents. *MedEdPORTAL*. 2017; 13:10555. https://doi.org/10.15766/mep_2374-8265.10555

4. Herlitz J, Bång A, Alsén B, Aune S. Characteristics and outcome among patients suffering from in-hospital cardiac arrest in relation to the interval between collapse and start of CPR. *Resuscitation*. 2002;53(1):21-27. https://doi.org/10.1016/S0300-9572(01)00485-3

5. Cooper S, Cade J. Predicting survival, in-hospital cardiac arrests: resuscitation survival variables and training effectiveness. *Resuscitation*. 1997;35(1):17-22. https://doi.org/10.1016/S0300-9572(97)00020-8

6. Grant EC, Marczinski CA, Menon K. Using pediatric advanced life support in pediatric residency training: does the curriculum need resuscitation? *Pediatr Crit Care Med*. 2007;8(5):433-439. https://doi.org/10.1097/01.PCC.0000282044.78432.0B

7. White JRM, Shugerman R, Brownlee C, Quan L. Performance of advanced resuscitation skills by pediatric housestaff. *JAMA Pediatrics*. 1998;152(12):1232-1235. https://doi.org/10.1001/archpedi.152.12.1232

8. Roy KM, Miller MP, Schmidt K, Sagy M. Pediatric residents experience a significant decline in their response capabilities to simulated life-threatening events as their training frequency in cardiopulmonary resuscitation decreases. *Pediatr Crit Care Med*. 2011;12(3):e141-e144. https://doi.org/10.1097/PCC.0b013e3181f3a0d1

9. Nadel FM, Lavelle JM, Fein JA, Giardino AP, Decker JM, Durbin DR. Assessing pediatric senior residents' training in resuscitation: fund of knowledge, technical skills, and perception of confidence. *Pediatr Emerg Care*. 2000;16(2):73-76. https://doi.org/10.1097/00006565-200004000-00001

10. Eppich WJ, Adler MD, McGaghie WC. Emergency and critical care pediatrics: use of medical simulation for training in acute pediatric emergencies. *Curr Opin Pediatr*. 2006;18(2):266-271. https://doi.org/10.1097/01.mop.0000193309.22462.c9

11. Ross JC, Trainor JL, Eppich WJ, Adler MD. Impact of simulation training on time to initiation of cardiopulmonary resuscitation for first-year pediatrics residents. *J Grad Med Educ*. 2013;5(4):613-619. https://doi.org/10.4300/JGME-D-12-00343.1

12. Friedman D, Zaveri P, O'Connell K. Pediatric mock code curriculum: improving resident resuscitations. *Pediatr Emerg Care*. 2010;26(7):490-494. https://doi.org/10.1097/PEC.0b013e3181e5bf34

13. van Schaik SM, Von Kohorn I, O'Sullivan P. Pediatric resident confidence in resuscitation skills relates to mock code experience. *Clin Pediatr*. 2008;47(8):777-783. https://doi.org/10.1177/0009922808316992

14. Andreatta P, Saxton E, Thompson M, Annich G. Simulation-based mock codes significantly correlate with improved pediatric patient cardiopulmonary arrest survival rates. *Pediatr Crit Care Med*. 2011;12(1):33-38. https://doi.org/10.1097/PCC.0b013e3181e89270

15. Hunt EA, Duval-Arnould JM, Nelson-McMillan KL, et al. Pediatric resident resuscitation skills improve after "rapid cycle deliberate practice" training. *Resuscitation*. 2014;85(7):945-951. https://doi.org/10.1016/j.resuscitation.2014.02.025

16. Sawyer T, Eppich W, Brett-Fleegler M, Grant V, Cheng A. More than one way to debrief: a critical review of healthcare simulation debriefing methods. *Simul Health*. 2016;11(3):209-217. https://doi.org/10.1097/SIH.0000000000000148

17. Phrampus PE, O'Donnell JM. Debriefing using a structured and supported approach. In: Levine AI, DeMaria S, Schwartz AD, Sim AJ eds. *The Comprehensive Textbook of Healthcare Simulation*. Springer; 2013:73-85. https://doi.org/10.1007/978-1-4614-5993-4_6

18. Cheng A, Rodgers DL, van der Jagt E, Eppich W, O'Donnell J. Evolution of the pediatric advanced life support course: enhanced learning with a new debriefing tool and web-based module for pediatric advanced life support instructors. *Pediatr Crit Care Med*. 2012;13(5):589-595. https://doi.org/10.1097/PCC.0b013e3182417709

19. Zaritsky A, Nadkarni V, Hazinski MF, et al. Recommended guidelines for uniform reporting of pediatric advanced life support: the pediatric Utstein style: a statement for healthcare professionals from a task force of the American Academy of Pediatrics, the American Heart Association, and the European Resuscitation Council. Writing Group. *Circulation*. 1995;92(7): 2006-2020. https://doi.org/10.1161/01.CIR.92.7.2006

Received: September 23, 2019
Accepted: March 30, 2020
Published: September 25, 2020

Copyright © 2020 Wing et al. This is an open-access publication distributed under the terms of the Creative Commons Attribution license.
